# A systematic review and quality assessment of individualised breast cancer risk prediction models

**DOI:** 10.1038/s41416-019-0476-8

**Published:** 2019-05-22

**Authors:** Javier Louro, Margarita Posso, Michele Hilton Boon, Marta Román, Laia Domingo, Xavier Castells, María Sala

**Affiliations:** 1 Department of Epidemiology and Evaluation, IMIM (Hospital del Mar Medical Research Institute), Barcelona, Spain; 2 Research Network on Health Services in Chronic Diseases (REDISSEC), Barcelona, Spain; 3 European Higher Education Area (EHEA) Doctoral Programme in Methodology of Biomedical Research and Public Health in Department of Pediatrics, Obstetrics and Gynecology, Preventive Medicine and Public Health, Universitat Autónoma de Barcelona (UAB), Bellaterra, Barcelona, Spain; 4 MRC/CSO Social and Public Health Sciences Unit, University of Glasgow, Glasgow, UK

**Keywords:** Epidemiology, Breast cancer, Epidemiology, Breast cancer, Epidemiology

## Abstract

**Background:**

Individualised breast cancer risk prediction models may be key for planning risk-based screening approaches. Our aim was to conduct a systematic review and quality assessment of these models addressed to women in the general population.

**Methods:**

We followed the Cochrane Collaboration methods searching in Medline, EMBASE and The Cochrane Library databases up to February 2018. We included studies reporting a model to estimate the individualised risk of breast cancer in women in the general population. Study quality was assessed by two independent reviewers. Results are narratively summarised.

**Results:**

We included 24 studies out of the 2976 citations initially retrieved. Twenty studies were based on four models, the Breast Cancer Risk Assessment Tool (BCRAT), the Breast Cancer Surveillance Consortium (BCSC), the Rosner & Colditz model, and the International Breast Cancer Intervention Study (IBIS), whereas four studies addressed other original models. Four of the studies included genetic information. The quality of the studies was moderate with some limitations in the discriminative power and data inputs. A maximum AUROC value of 0.71 was reported in the study conducted in a screening context.

**Conclusion:**

Individualised risk prediction models are promising tools for implementing risk-based screening policies. However, it is a challenge to recommend any of them since they need further improvement in their quality and discriminatory capacity.

## Background

Mammography screening has been associated with a reduction in breast cancer mortality and therefore organised breast cancer screening programmes using mammography have been well established worldwide.^1–4^ Although there is not a single consensus, current screening programmes generally recommend biennial or triennial screening in Europe and annual or biennial screening in the US with variations in the recommended targeted age.^2–5^ These recommendations usually consider age as the sole risk factor leading women to be invited for screening from age 40–50 until age 70–74, depending on the programmes.

The likelihood that a woman will benefit from screening mammography depends on her risk for developing clinically significant breast cancer in her lifetime. Taking individual risk factors beyond age into account should enable the classification of women into groups at varying risk of breast cancer. Personalised risk-based screening going beyond the current ‘one-size fits all' recommendation may increase the effectiveness and benefit-harm balance of breast cancer screening. Individualised risk prediction models for breast cancer are a key element to develop risk-based screening approaches since they are designed to quantify the risk that can predict whether an individual woman would develop breast cancer in a defined period.^6^

A number of risk prediction models that include classical risk factors are commonly used in clinical contexts.^7^ However, organised screening programmes do not use these models routinely. One reason for not including these models in screening context is the high uncertainty with regards to its applicability in screening settings. Also, the emergence of new risk prediction factors such as the expression of single nucleotide polymorphisms (SNPs) needs to be appropriately summarised before recommending one of the models into screening practice.

Like any other source of information, risk prediction models have limitations that should be evaluated before using them. A rigorous risk of bias assessment of the existing individualised risk models is needed to clarify the overall quality and applicability of each model. Therefore, the aim of this systematic review is to update the existing evidence, conduct a critical appraisal and risk of bias assessment and summarise the results of the individualised risk models which are used to estimate the risk of breast cancer in women in the general population.

## Methods

### Data sources and searches

We performed a systematic review of the literature following the standard Cochrane Collaboration methods^8^ and adhering to the PRISMA statement reporting recommendations.^9^ A predetermined review protocol was registered (CRD42018089842) in the PROSPERO database (date of registration 1 March 2018). The Patient, Intervention, Comparison, Outcomes (PICO) question of this systematic review is the following: Should individualised breast cancer risk prediction models vs. no risk prediction models be used to develop risk-based screening approaches for women in the general population?

We retrieved relevant literature by using a combination of controlled vocabulary and keyword search terms in the following databases: (i) Medline (accessed through PubMed); (ii) The Cochrane Library; and (iii) EMBASE (accessed through Ovid). Terms related to breast cancer recurrence were excluded in order to avoid retrieving citations out of the scope of this systematic review. We adapted the search algorithms to the requirements of each database and used validated filters to retrieve systematic reviews and primary studies as needed. We reviewed references of included studies that could potentially fulfil our eligibility criteria. The detailed search strategy is reported in Supplementary table 1.

We searched primary studies of individualised breast cancer risk models searching each database from its inception up to February 2018.

### Study selection

Eligible studies were those published in English that reported a model to estimate the individualised risk of breast cancer in women in the general population. We included models that assessed more than one risk factor and reported the quantitative characteristics of the risk prediction model. If multiple publications were based on the same individualised risk model, the most extensive report of the model in terms of risk factors reported was chosen. We excluded external validation studies that replicated previous models without adding any additional information such as a new design for collecting the inputs data, modifications on the risk factors or the risk model method.

Articles identified from the search were loaded into EndNote X7.7.1 for Windows (2008, Version 12.0.4) and duplicates were removed.

### Data extraction and quality assessment

**Fig. 1:**
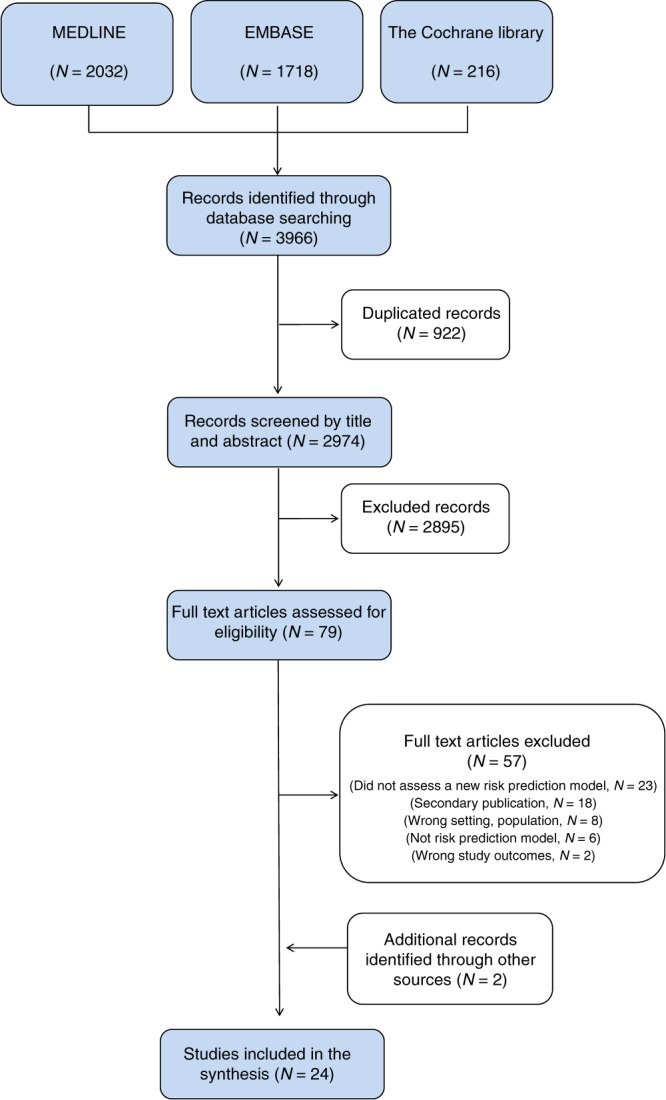
PRISMA flowchart

One reviewer screened the search results based on title and abstract, and a second reviewer performed a quality check of the study screening by reviewing 20% of the references. Two reviewers independently confirmed eligibility based on the full text of the relevant articles. In case of disagreement between researchers, the inclusion of studies was determined by consensus. We reported the result of this process with a PRISMA flowchart (Fig. 1).

We used a predefined form to extract the following information from included studies: author, publication date, country, study design, the name of the model if available, sample characteristics, sample size, type of breast cancer, the method of analysis, and validation of the model. Data abstraction was conducted by one reviewer and checked by another.

Two reviewers carried out the assessment of the risk of bias independently and final quality assessment was based on consensus. We used the ISPOR-AMCP-NPC Questionnaire^10^ to assess the relevance and credibility of each risk prediction study and the following sources of limitations: (i) internal and external validation; (ii) bias due to the study design for risk estimates; (iii) limitations in data inputs; (iv) appropriateness of the model analysis; (v) reporting bias; (vi) interpretation bias; and (vii) conflict of interest. The risk of bias for each domain was rated as low, high or unclear. For systematic reviews we used the AMSTAR 2 critical appraisal tool.^11^

### Data synthesis and analysis

We evaluated the model validation by assessing both the discriminative power and the calibration accuracy estimated for the women in the general population. When available in the included publication, we extracted the area under the receiver operating characteristic curve (AUROC), the net reclassification index (NRI) and the expected observed (E/O) ratio. The NRI was not included in the tables because it was only reported in 2 out of 24 articles. The characteristics of the included models and the risk prediction outcomes reported preclude the possibility to pool data across studies. Therefore, a narrative synthesis has been conducted. Key study characteristics, validation and accuracy of individual risk models, and methodological quality are described in tables and summarised in a narrative manner. Results are presented according to the original model that they reported.

## Results

### Study inclusion

The database searches for primary studies retrieved 2974 citations, of which 79 were considered potentially relevant. These 79 studies were screened in full text. We found a systematic review of Anothaisintawee et al.,^7^ which we used as a source of primary studies. In addition, two studies were included after a manual inspection of papers’ references.^12,13^ After the full text was checked, 24 studies^12–35^ met the inclusion criteria and were considered in the evidence synthesis. Details about study inclusion with reasons for exclusion are described in the flow-chart (Fig. 1), and a list of references to excluded studies is provided in Supplementary table 2.

### Characteristics of the included studies

The included studies can be grouped according to the risk model that they reported, the Breast Cancer Risk Assessment Tool (BCRAT), the Breast Cancer Surveillance Consortium (BCSC), the Rosner & Colditz model, the International Breast Cancer Intervention Study (IBIS), and other original models. The study by Zhang et al.^13^ is included in two of the groups (BCRAT and Rosner & Colditz models) because it provides information of both models and presents its results separately. A brief summary of the 24 included studies is presented in Table 1 and the extended characteristics in Supplementary table 3.

- Breast Cancer Risk Assessment Tool ‘BCRAT’ model. This model was first published in the United States in 1989 assessing age, family history of breast cancer, age at first birth, menarche, and previous biopsies as risk factors for predicting individualised breast cancer risk.^22^ After this first publication, eight studies were identified that were based on BRCAT model but modified the data collection design, assessed additional risk factors or changed the statistical method. In addition to the five risk factors proposed in 1989, other variables such as body mass index (BMI), weight, hormone replacement therapy (HRT), alcohol consumption, physical activity, diet, breast density, atypical hyperplasia, breast inflammatory disease, parity, a polygenic risk score or hormones information have been included in updated versions (Table 1).^13,14,16,17,20,23,25,26,30^
- Breast Cancer Surveillance Consortium ‘BCSC’ model. One relevant variation of the BCRAT model opens the path to the emergence of the BCSC model first published by Tice et al. in 2008 in the United States.^31^ In this study, Tice et al. used data from a cohort to create an individualised risk prediction model that combines age, family history, previous biopsies, breast density, and ethnicity. The BCSC model has been further evaluated by other authors^12,24,29,32^ and it currently includes previous benign breast diseases and polygenetic risk score using SNPs as risk factors (Table 1).
- Rosner & Colditz model. Parallel to the BCSC model, another model based on the ‘Nurses' Health Study’ cohort developed by Rosner & Colditz in 1996 was also developed in the United States. This model currently includes 11 risk factors: age, menarche, menopause, age at first birth, age at subsequent births, previous benign breast disease, HRT, family history, weight, BMI, alcohol consumption, and oestradiol levels.^18,19,27,28^ In the same way as in the BCRAT, Zhang et al.^13^ analysed this model adding breast density, a polygenic risk score and endogenous hormones as risk factors.
- International Breast Cancer Intervention Study ‘IBIS’ model. The IBIS model^33^ includes genetic information adding the BRCA genes and a hypothetical susceptibility gene.
- Other models. Four studies reporting different models were also identified.^15,21,34,35^ Apart from the above-mentioned risk factors, the models also assessed other variables such as abortion, breastfeeding, height, and previous mammography results. Particularly relevant is the Eriksson model^21^ since it was the only one targeted to the screening population. In this study, the authors included risk factors that were available at mammography screening examination: age, BMI, HRT, family history, menopause, breast density, and presence of microcalcifications and/or masses in the screen-mammogram.

**Table 1:** Summary of included studies

Study ID	Targeted population	Risk factors (number of categories)	Discriminatory accuracy (AUROC)^a^	Calibration (E/O ratio)^a^
BCRAT model				
Banegas 2017	Hispanic, 25–79 years	Age (2), Menarche (3), Previous biopsies (2), Age at first birth (3), First degree breast cancer (2)	US-born: 0.56 Foreign-born: 0.62	US-born: 0.93^b^ Foreign-born: 1.52^b^
Boyle 2004	Caucasian, 20–74 years	Age (n), Menarche (3), Age at first birth (3), First degree breast cancer (2), BMI (3), Alcohol (3), Physical activity (3), HRT (2), Diet beta-carotene/vitE (5), Diet fruits/vegetables (5)	0.6	1.03^b^
Chen 2006	Caucasian, 35–74 years	Age (2), Weight (6), Breast density (5), Menarche (3), Previous biopsies (3), Age at first birth (4), First degree breast cancer (3), Atypical hyperplasia (2)	None	None
Decarli 2006	Caucasian, 20–74 years	Age (2), Menarche (3), Previous biopsies (3), Age at first birth (4), First degree breast cancer (3)	0.59	0.96
Gail 1989	Caucasian, 20–79 years	Age (2), Menarche (3), Previous biopsies (3), Age at first birth (4), First degree breast cancer (3)	None	None
Gail 2007	African-American, 35–64 years	Age (2), Menarche (3), Previous biopsies (3), Age at first birth (4), First degree breast cancer (3)	0.56	0.93^b^
Matsuno 2011	Asian, 20–55 years	Age (2), Menarche (3), Previous biopsies (3), Age at first birth (4), First degree breast cancer (n), Ethnicity (6)	0.61	0.85^b^
Novotny 2006	Multiple ethnicities, 23–84 years	Age (2), Menarche (3), Previous biopsies (3), Age at first birth (4), First degree breast cancer (3), First degree family history of cancer (5), Parity (n), Breast inflammatory disease (2)	None	None
Tice 2005	Multiple ethnicities, older than 35 years	Age (2), Menarche (3), Previous biopsies (3), Age at first birth (4), First degree breast cancer (3), Breast density (4)	0.68	None
Zhang 2018	Caucasian 30–64 years	Age (2), Menarche (3), Previous biopsies (3), Age at first birth (4), First degree breast cancer (2), Polygenic Risk Score(n), Mammographic density (n), Estrone Sulphate (n), Testosterone (n), Prolactin (n)	0.65	None
Breast Cancer Surveillance Consortium ‘BCSC’ model				
Kerlikowske 2015	Multiple ethnicities, 35–74 years	Age (n), Ethnicity (6), First degree breast cancer (2), Previous biopsies (2), Changes in breast density (16)	0.64	5-years: 0.98 10-years: 0.95
Shieh 2016	Multiple ethnicities. Age was not specified	Age (n), Ethnicity (6), First degree breast cancer (2), Previous biopsies (2), Breast density (4), Polygenetic risk score (n), BMI (n)	0.65	None
Tice 2008	Multiple ethnicities, 35–84 years	Age (n), Ethnicity (4), First degree breast cancer (2), Previous biopsies (2), Breast density (BI-RADS)	0.66	1.03
Tice 2015	Multiple ethnicities, 35–74 years	Age (n), Ethnicity (4), First degree breast cancer (2), Breast density (4), Benign breast disease (6)	0.67	5-years: 1.04 10-years: 1.05
Vachon 2015	Multiple ethnicities. Age was not specified	Age (n), Ethnicity (6), First degree breast cancer (2), Previous biopsies (2), Breast density (4), Polygenetic risk score (*n*)	0.69	None
Rosner & Colditz model based on the ‘Nurses' Health Study’				
Colditz 2000	Caucasian, 30–64 years	Age (*n*), Menarche (*n*), Age at first birth (*n*), Menopause (*n*), Age at subsequent births (*n*), Benign breast disease (2), HRT (*n*), First degree breast cancer (2), Weight (*n*), BMI (*n*), Alcohol (*n*)	None	None
Colditz 2004	Caucasian, 30–64 years	Age (*n*), Menarche (*n*), Age at first birth (*n*), Menopause (*n*), Age at subsequent births (*n*), Benign breast disease (2), HRT (*n*), First degree breast cancer (2), Weight (*n*), BMI (*n*), Alcohol (*n*)	ER+/PR+: 0.64 ER−/PR−: 0.61	None
Rosner 1996	Caucasian, 30–64 years	Age (*n*), Menarche (*n*), Age at first birth (*n*), Menopause (*n*), Age at subsequent births (*n*)	None	None
Rosner 2008	Caucasian, 30–64 years	Age (*n*), Menarche (*n*), Age at first birth (*n*), Menopause (*n*), Age at subsequent births (*n*), Benign breast disease (2), HRT (*n*), First degree breast cancer (2), Weight (*n*), BMI (*n*), Alcohol (*n*), Estradiol levels (*n*)	0.65	None
Zhang 2018	Caucasian 30–64 years	Age (*n*), Menarche (*n*), Age at first birth (*n*), Menopause (*n*), Age at subsequent births (*n*), Benign breast disease (2), HRT (*n*), First degree breast cancer (2), Weight (*n*), BMI (*n*), Alcohol (*n*), Polygenic Risk Score(*n*), Mammographic density (*n*), Estrone Sulphate (*n*), Testosterone (*n*), Prolactin (*n*)	0.68	None
IBIS model				
Tyrer 2004	Multiple ethnicities. Age was not specified	Age (*n*), Gen phenotype (6), Family history (*n*, relationship, age), Menarche (*n*), Age at first birth (5), Menopause (*n*), Atypical Hyperplasia (2), Lobular carcinoma in situ (2), Height (3), BMI (5)	None	None
Other original models				
Barlow 2006	Multiple ethnicities, 35 to 84 years	Age (9), Age at first birth (4), Ethnicity (6), Menopause (2), First degree breast cancer (5), Previous biopsies (4), Breast density (5), HRT (3), BMI (5), Previous false positive or true negative screen result (2), Menopausal status (3)	Pre menopause: 0.63 Post menopause: 0.62	Pre menopause: 1.00 Post menopause: 1.01
Eriksson 2017	Caucasian, 40–74 years	Age (7), BMI (n), HRT (2), Breast cancer family history (2), Menopause (2), Breast density (4), Microcalcifications (5), Mases (n)	0.71	None
Ueda 2003	Asian women. Age was not specified.	Age (n), Menarche (3), Age at first birth (5), BMI (2), Breast cancer family history (2)	None	None
Tyrer 2004	Multiple ethnicities. Age was not specified	Age (*n*), Gen phenotype (6), Family history (*n*, relationship, age), Menarche (*n*), Age at first birth (5), Menopause (*n*), Atypical Hyperplasia (2), Lobular carcinoma in situ (2), Height (3), BMI (5)	None	None
Wang 2014	Asian, 35–70 years	Age (7), Menarche (2), Previous biopsies (2), Age at first birth (2), First degree breast cancer (2), Breastfeeding (2), Abortion (2)	0.64	None

^a^Discriminatory and calibration accuracy values represents the statistics published in the original articles for the general population Subgroup values are not reported here

^b^The original publication reported the Observed/Expected ratio. E/O ratios were calculated based on the original information. *AUROC* area under the receiver operating characteristic curve. *E/O* expected/observed, *BMI* body mass index, *HRT* hormone replace treatment, *ER* oestrogen receptor, *PR* progesterone receptor, *BI-RADS* Breast Imaging Reporting and Data System. (n): continuous variable in model

### Discriminatory accuracy

**Fig. 2:**
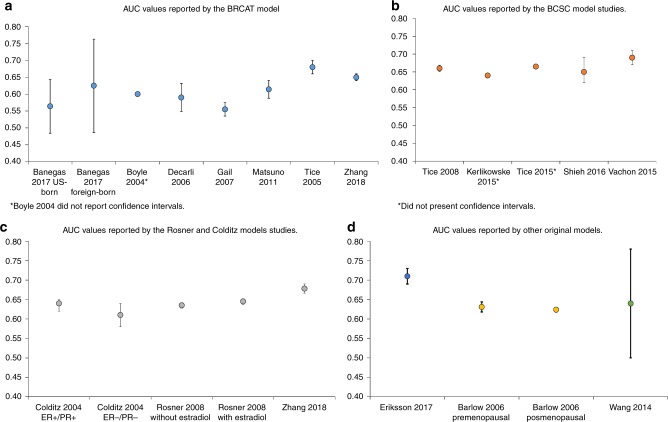
Area under the ROC curve (AUROC) and Confidence Intervals reported by the included studies. **a** AUROC values reported by the BCRAT model studies. **b** AUROC values reported by the BCSC model studies. **c** AUROC values reported by the Rosner & Colditz model studies. **d** AUROC values reported by other original models

Fifteen out of the 24 studies reported the discriminatory accuracy as the AUROC (Table 1 and Fig. 2).

- BCRAT model. The first BCRAT model publication did not report the AUROC, however, later publications of this model reported a range that varied from 0.56 to 0.68. The three publications that included the original risk factors, age, family history of breast cancer, age at first birth, menarche, and previous biopsies, reported low AUROC values, 0.56 to 0.62.^14,20,23^ Similarly, the AUROC reported by Boyle et al.^16^ and Matsuno et al.^25^ were 0.60 and 0.61, although these authors added BMI, HRT, alcohol, physical activity and diet, and ethnicity into the model. Zhang et al.^13^ with the new variables reach an AUROC of 0.65 and Tice et al.^30^ reported in 2005 a higher AUROC value of 0.68 which was obtained just adding breast density to the original five risk factors (Table 1). Zhang et al.^13^ also reported the NRI to validate that his model improved the previous ones with a result of 8%.
- BCSC model. The published value of the AUROC for the BCSC model was moderate, ranging from 0.64 to 0.69. Tice et al. included age, family history, previous biopsies, breast density reported by the Breast Imaging Reporting and Data System (BI-RADS), and ethnicity into the model in 2008 and obtained a value of 0.66 for the AUROC.^31^ Instead of BI-RADS, Kerlikowske et al. assessed changes in breast density obtaining a similar result, 0.64.^24^ Using previous benign breast disease, Tice et al. obtained a slightly higher AUROC value of 0.67 in 2015.^32^ More recently, in 2015 and 2016, Vachon et al.^12^ added to the model a polygenic risk score and Shieh et al.^29^ a combination between a polygenic risk score and BMI reporting a value of 0.69 and 0.65 for the AUROC respectively (Table 1). Vachon et al.^12^ also demonstrated the improvement of discriminatory accuracy estimating the NRI with a positive result of 11%.
- Rosner & Colditz model. The discriminatory accuracy of this model varied from 0.61 to 0.68. The authors assessed age, family history, age at first birth, menarche, BMI, benign breast disease, menopause, HRT, age at subsequent births, alcohol, and weight. They obtained an AUROC of 0.64 and 0.61 for ER + /PR + and ER-/PR- tumours, respectively.^19^ The addition of oestradiol levels to the model was tested by Rosner et al. who obtained a 0.65 AUROC value in 2008.^28^ Finally the addition of a polygenic risk score, mammographic density and endogenous hormones by Zhang et al.^13^ reached a 0.68 AUROC value (Table 1) and obtained an improvement of the discriminative accuracy also reflected in a NRI of a 9.5%.
- IBIS model. The IBIS model original paper^33^ does not include any validation and does not present the AUROC. Nevertheless, it has been externally validated showing an AUROC of 0.57 which increases to 0.61 when adding mammographic density.^36^
- Other models. Overall, the AUROC values of these models were not higher than those shown by the above-mentioned models, varying from 0.62 to 0.64, although they included a large number of risk factors. However, the model reported by Eriksson et al.^21^ did show an AUROC of 0.71 that was the highest AUROC value identified in this systematic review (Table 1). This model, in addition, is the only one that estimates a 2-year risk, while the rest of models estimate the risk at a longer time horizon. This could explain the difference in AUROC values since it becomes more difficult to predict risk as the time horizon increases.

### Calibration accuracy

Nine out of the 24 studies reported the calibration accuracy as the E/O ratio (Table 1).

- BCRAT model. Of the 10 studies derived of the BCRAT model, five reported the calibration accuracy. Banegas et al.^14^ presented heterogeneous results depending on the provenance of the population, reporting an E/O ratio of 0.93 for US-born and 1.52 for foreign-born women. Although Matsuno et al.^25^ added new variables to the original BCRAT model, the E/O ratio was 0.85, which was the lowest of the group, whereas the other studies published E/O ratios that varied from 0.93 to 1.03^16,20,23^ (Table 1).
- BCSC model. Tice et al. published in 2008 a value of 1.03 for the E/O ratio when looking at 5-year risk.^31^ Using previous benign breast disease, they obtained a similar result in 2015, with an E/O ratio of 1.04 for 5-year risk and 1.05 for 10-year risk.^32^ When Kerlikowske et al. assessed changes in breast density the ratio decreased obtaining a 0.98 for 5-year risk and 0.95 for 10-year risk.^24^ The studies of Vachon et al. and Shieh et al. did not present validation regarding the calibration accuracy of the model (Table 1).
- Rosner & Colditz model. Of the five studies based on the Rosner & Colditz model,^13,18,19,27,28^ none of them reported calibration accuracy statistics of their models for the women in the general population.
- IBIS model. The IBIS model original paper^33^ does not report any calibration statistic. Nevertheless, other articles have validated it showing an E/O ratio of 1.67.^36^
- Other models. The study Barlow et al.^15^ was the only one that reported calibration accuracy and presented the closest E/O ratio to one of all the studies included in this review taking values of 1.00 and 1.01 for pre and post-menopausal status respectively (Table 1).

### Quality assessment

**Fig. 3:**
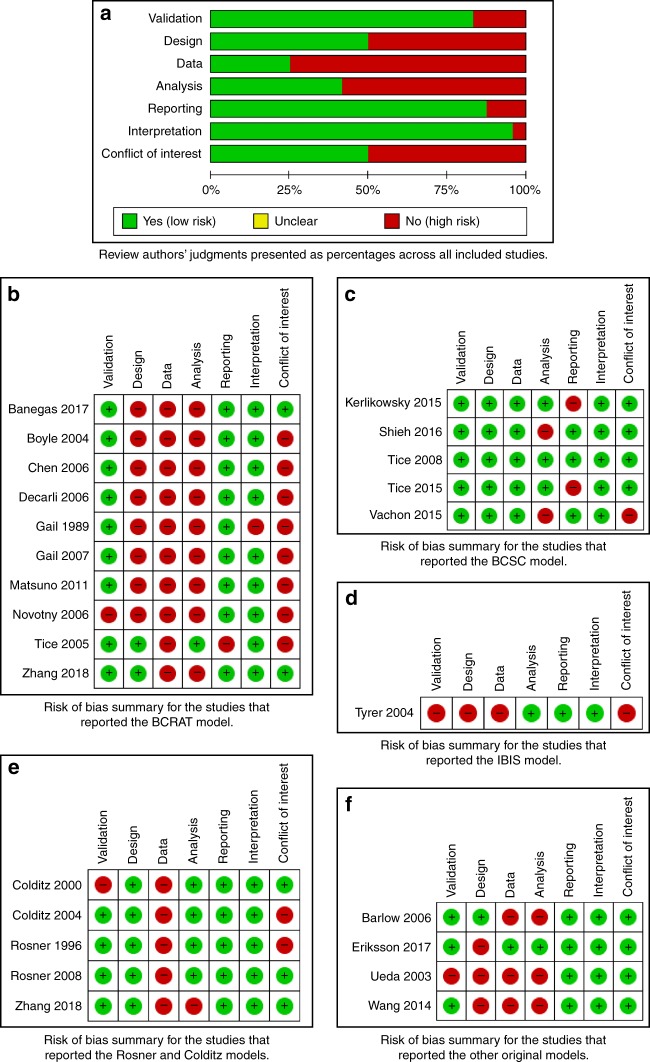
Risk of bias summary: review authors’ judgments about each risk of bias item for each included study. **a** Review authors’ judgments presented as percentages across all included studies. **b** Risk of bias summary for the studies that reported the BCRAT model studies. **c** Risk of bias summary for the studies that reported the BCSC model studies. **d** Risk of bias summary for the studies that reported the Rosner & Colditz model studies. **e** Risk of bias summary for the studies that reported other original models

The quality of the included studies was moderate due to some limitations in the discriminative power, study design, and data inputs. The studies did not show important limitations with regards to the validation, appropriateness of the model analysis, reporting or interpretation of the results (Fig. 3). A summary of the risk of bias assessment per each source of limitation is presented here and the detailed appraisal and judgements in Supplementary table 4.

### Internal and external validation

Ten studies^14–17,20,23,25,26,30,31^ validated their models by comparing the results with those published by Gail et al.,^22^ three studies^24,29,32^ compared with Tice et al.,^31^ one^21^ compared with both Gail et al.^22^ and Tyrer et al.,^33^ one^13^ compared with both Gail et al.^22^ and the results of a Rosner & Colditz model external validation^37^ and three studies did not report the model validation in the primary articles.^19,22,34^ Six studies assessed internal validation with a sample of the population that generated data for the model,^15,16,24,29,31,32^ and four with an external population.^14,20,23,25^ Despite not having reported the external validation in the primary articles, the Rosner & Colditz model^18,19,27,28^ reported external validation in a subsequent article mentioned before.^37^ Nine studies used the expected/observed event ratio to measure the calibration accuracy of the model.^14–16,20,23–25,29,31^

### Bias due to the study design

Thirteen studies used a case-control design to obtain breast cancer risk estimates,^12–14,16,17,20–23,25,26,29,34^ five studies used prospective cohorts,^15,18,19,27,28^ and four models used retrospective cohorts.^24,30–32^ The study of Wang et al.^35^ and the study of Tyrer et al.^33^ used risk estimates obtained from a systematic review of the literature.

### Limitations of data inputs

Sixteen studies obtained most of the input parameters from self-reported questionnaires.^13–20,22,23,25–28,30,34^ The study of Matsuno et al.^25^ also imputed ethnicity for women with missing data.

### Appropriateness of the model analysis

Thirteen studies^12–17,20,22,23,25,26,29,34^ used logistic regression to estimate the risk of having breast cancer according to the assessed risk factors, five used proportional hazard Cox models,^21,24,30–32^ four used Poisson regression models,^18,19,27,28^ and the other two studies used risk estimates obtained from a systematic review of the literature.^33,35^

### Reporting bias

Twenty one studies reported all relevant and necessary information for the model creation.^12–23,25–29,31,33–35^ Conversely, a critical lack of information was found in the other three studies.^24,30,32^

## Discussion

### Summary of main results

This systematic review included 24 studies that aimed to estimate the individual risk of developing breast cancer in women in the general population. Twenty studies were based on four specific risk models (the BCRAT, the BCSC, the Rosner & Colditz and the IBIS model),^16–20,22–33^ whereas four studies used other original models.^15,21,34,35^ The most extensively used were the BCRAT, IBIS and the BCSC models. The number of risk factors included in the models ranged from five to 18. Other than age, which was the only risk factor present in all models, the BCRAT model also included family history, age at first birth, menarche, and previous biopsies. Breast density, benign breast disease, and polygenetic score were predominant in the BCSC model. Although during the last decade the models have shown improvements in their discriminatory accuracy, it remains at best moderate with a maximum AUROC value of 0.71 reported by Eriksson et al.^21^ The calibration accuracy was very heterogeneous ranging from 0.85 to 1.52. Furthermore, the quality of the studies was not high due to limitations in the discriminative accuracy, study design, and data inputs.

### Agreements and disagreements with other reviews

In this systematic review, we found that the number of individualised breast cancer risk prediction models has increased steadily over the past three decades. This finding is in agreement with the narrative overview published by Cintolo-Gonzalez et al. in 2017,^38^ and it updates the results of a previous systematic review published by Anothaisintawee et al. in 2012.^7^ In contrast to these reviews, however, our aim was to provide innovative information regarding the quality of the identified prediction models. Thus, we have identified and rigorously analysed the strengths and limitations of 24 individualised models in order to adjust our conclusions to the quality of the evidence.

We have identified two new trends with regards to the use and development of the models, which are the increased use of the BCSC model and the inclusion of common genetic variation in the prediction models. As compared to the information published in the review of Anothaisintawee et al.,^7^ we found that in contrast to the BCRAT and Rosner & Colditz models that were the most frequently cited models up to 2010^7^ the BCSC model has concentrated the attention of several authors during the last five years, although its discriminatory accuracy has not dramatically improved. Second, none of the models in the review of Anothaisintawee et al.^7^ included genetic information as a risk factor. By contrast, we have identified four models including genetic information: the IBIS model^33^ that includes genetic phenotype in their updated version, the BCSC model that includes a polygenetic score in both 2015^12^ and 2016^29^ publications, as well as the article by Zhang et al. that added a polygenic risk score to both the BCRAT and the Rosner & Colditz models.^13^

Most of the included studies reported the AUROC to determine the probability that a randomly chosen woman with disease would be correctly categorised as higher risk compared to a randomly chosen woman without disease. The discriminatory accuracy estimate does not express whether the model is more or less accurate in predicting the risk of specific individuals but measures the capacity of the model to determine which women are at higher/lower risk for developing breast cancer. Thus, both calibration accuracy and discriminatory accuracy should be assessed. Contrary to what is expected, we found that authors reported the E/O ratio only in less than half of the included studies. In addition to the AUROC value, the studies of Zhang et al. and Vachon et al.^12,13^ also reported an improvement in the net reclassification index (NRI) of the BCRAT, and Rosner & Colditz models, as well as in the BCSC model, respectively.

Overall, the information provided by the AUROC and the E/O ratio was consistent suggesting that the included models have moderate discriminatory accuracy and calibration accuracy when applied to the women in the general population. Nevertheless, it must be taken into account that despite the great importance of validation in terms of AUROC and E/O ratio, the presence of low values of AUROC or clearly different from 1 values of the E/O ratio does not mean that these models are useless. On the contrary, models are clinically useful even with moderate AUROC since they can reclassify individuals at the extremes of risk.^39^ Thus, the verdict on risk models should not be based solely on these estimators. Instead, they need to be prospectively evaluated in clinical trials. In fact, there are currently two very large randomised trials assessing risk-based screening strategies. Both of them are using individualised models. Both the IBIS and the BCSC models are being tested in the European trial MyPeBS (My Personalised Breast Screening).^40^ Also, the BCSC model is being tested in the US WISDOM trial (Women Informed to Screen Depending On Measures of risk).^41^

### Applicability and completeness of evidence

The distribution of risk factors in such different populations may affect the applicability of the models to different contexts. The fact that different subtypes of breast cancer may have different genetic markers is widely accepted.^42^ These differences, the nature of breast cancer itself and its low incidence may condition a low discriminatory accuracy of a model. In other words, in the general population, there is a low probability of having breast cancer (even in the highest risk group). This low probability may mean that the discriminatory power of a breast cancer risk model won’t be as high as a risk model targeted to other common diseases such as cardiovascular events, for instance. Another potential limitation in the applicability in the screening context is the completeness and the number of included risk factors, which ranged from five to 18. Nevertheless, some potentially relevant risk factors such as genetic markers have been only included in few models. Recent studies^43,44^ have shown that adding genetic information as a risk factor can increase the discriminative accuracy of the different models which opens the line for further evaluation. An evaluation that should first assess the calibration of these models in prospective cohort studies.

Overall, women are usually screened using mammography. Particularly in Europe, most programmes invite women for screening every 2 years.^2^ The presence of some mammographic features in these screening mammograms may be related to the risk of developing breast cancer, as has been recently pointed out by some authors.^21,45^ Only one of the 24 models identified in this systematic review included microcalcifications and masses found at mammography as risk factors in the model.^21^ Time-changing variables such as radiological variables may not be as stable as personal history. However, in a screening context, this information is especially relevant because it is easily available from previous screening examinations.

### Quality of the evidence

We found variability in the design of the studies that were used to obtain the cancer risk estimates. Notably, the study design used in the BCSC model was a cohort, which is a robust epidemiology design that allows developing and validating prediction models. Another frequently used design was the case-control study, nested or not. Contrary to the cohort study, time-changing variables may not be well obtained in case-control studies.

Regarding the external validation, the models showed some limitations given that few of them were further evaluated in different contexts. As far as we know, there are numerous scientific publications reporting external model validation in different settings and countries. These studies may help to understand the performance of a model in a specific context, but this issue was out of the scope of our review and, therefore, we have not included external validation studies. As an example of the relevance of these studies, we can inform that the BCRAT model has more than 50 articles informing the external validation of these models in different countries.^46^ The Rosner-Colditz model has also been validated in several studies, one of the most complete validations being the one performed in 2013 by the authors themselves.^37^ On the other hand, we found that although the Eriksson et al.^19^ model reports the highest AUC (0.71), this model has not been externally validated, which increases the uncertainty about its applicability.

Also, there were limitations in data inputs, mostly due to the fact that in several models the information was provided by self-reported questionnaires that may affect the accuracy of the results. Finally, there is a limitation when comparing the AUROC or E/O ratio across the models given that there is great heterogeneity amongst them. The models were targeted to different populations, included different sets of risk factors, and often used different methodologies. We have taken into account all these variations and presented the results by model categories.

### Potential biases in the review process

This systematic review was limited to studies published in English and did not involve an active search for grey literature, which is literature that is not formally published in sources such as books or journal articles. Therefore, some models may not have been identified. However, since we have conducted a comprehensive literature search in Medline, EMBASE and The Cochrane Library, we estimate that the loss of information due to the study selection criteria is low. Some key genetically oriented models, such as BOADICEA^47^ and BRACAPRO^48^ were not included in this review because they are aimed at high risk women and not useful for women in the general population in the screening context. Full-text screening and data abstraction process were performed by two researchers, which increase the quality of the review process. Moreover, as far as we know, this is the first review assessing the risk of bias of the identified risk prediction models.

## Conclusions

The development of individualised breast cancer risk prediction models has increased over the last three decades, but the improvements in both the discriminatory power and calibration accuracy are still limited. Despite the time that has passed since the first model was published and a large number of available publications, only one model addressed to women attending a population-based screening programme^21^ was identified. Currently, it is still a challenge to recommend any of the models as the standard for predicting individual risk in screening context. However, the models have been updated by adding new variables, such as common genetic variation or radiologic variables and have shown improvements in their quality as well as in their discriminative accuracy. These new variables need further evaluation to confirm its promising impact in the prediction capacity to propose personalised strategies for breast cancer screening.

## Supplementary information

Supplementary data

## Data Availability

The datasets analysed during the current study are publicly available from the corresponding author.
