# Supplementary material for: A systematic review and quality assessment of individualised breast cancer risk prediction models
Source: Br J Cancer. 2019 May 22;121(1):76–85. doi: 10.1038/s41416-019-0476-8 (PMC6738106; doi:10.1038/s41416-019-0476-8)
Supplement: Supplementary file 1 — Supplementary data [file 41416_2019_476_MOESM1_ESM.docx]

**Supplementary table 1. Search strategy**

| **MEDLINE**  (Through  PubMed)  **Search date**  February 19^th^, 2018 | #1 Search Search ("Breast Neoplasms"[Mesh] OR ((breast[Title/Abstract]) AND (neoplasm[Title/Abstract] OR neoplasms[Title/Abstract] OR cancer[Title/Abstract] OR cancers[Title/Abstract] OR tumor[Title/Abstract] OR tumors[Title/Abstract] OR carcinoma[Title/Abstract] OR carcinomas[Title/Abstract])))  #2 Search ("Risk"[Mesh] OR (risk[Title/Abstract] OR risks[Title/Abstract]))  #3 Search ("Models, Theoretical"[Mesh] OR model*[Title/Abstract])  #4 Search (predict*[Title/Abstract] OR assess*[Title/Abstract] OR estimat*[Title/Abstract/Abstract])  #5 Search (individual*[Title/Abstract] OR personal*[Title/Abstract] OR particular*[Title/Abstract])  #6 Search #1 AND #2 AND #3 AND #4 AND #5 2278  #7 Search "Recurrence"[Mesh]  #8 Search (recurrence[Title/Abstract] OR recurrences[Title/Abstract] OR relapse[Title/Abstract] OR relapses[Title/Abstract] OR recrudescence[Title/Abstract] OR recrudescences[Title/Abstract])  #9 Search #7 OR #8 479846  #10 Search #6 not #9 **2032** |
| --- | --- |
| **EMBASE**  (Through EbscoHost)  **Search date**  February 19^th^, 2018 | 1 breast cancer/ or breast tumor/ or breast carcinoma/  2 (breast and (cancer or cancers or neoplasm or neoplasms or tumor or tumors or carcinoma or carcinomas)).ab,ti.  3 1 or 2  4 exp risk/  5 (risk or risks).ab,ti.  6 4 or 5  7 exp model/  8 model*.ab,ti.  9 7 or 8  10 (predict* or assess* or estimat*).ab,ti.  11 (individual* or personal* or particular*).ab,ti.  12 3 and 6 and 9 and 10 and 11 2994  13 recurrent disease/  14 (recurrence or recurrences or recurrent or relaps* or recrudescence or recrudescences).ab,ti.  15 13 or 14  16 12 not 15 2538  17 limit 16 to conference abstracts  18 16 not 17 **1718** |
| **The Cochrane Library**  **Search date**  February 19^th^, 2018 | #1 MeSH descriptor: [Breast Neoplasms] explode all trees  #2 breast: ti,ab,kw and cancer or cancers or neoplasm or neoplasms or tumor or tumors or carcinoma or carcinomas:ti,ab,kw (Word variations have been searched)  #3 #1 or #2 26441  #4 MeSH descriptor: [Risk] explode all trees  #5 risk or risks:ti,ab,kw (Word variations have been searched)  #6 #4 or #5 162535  #7 MeSH descriptor: [Models, Theoretical] explode all trees  #8 model*:ti,ab,kw (Word variations have been searched) 934  #9 #7 or #8 94795  #10 predict* or assess* or estimat*:ti,ab,kw (Word variations have been searched) 399724  #11 individual* or personal* or particular*:ti,ab,kw (Word variations have been searched) 117842  #12 #3 and #6 and #9 and #10 and #11 **216** |

**Supplementary table 2. List of excluded studies**

| **Did not assess a new risk prediction model (applied an old model to a different population), N= 23** | | |
| --- | --- | --- |
| **Study ID** | **References** | **Source** |
| 1. Al Otaibi 2017 | Al Otaibi HH. Breast Cancer Risk Assessment Using the Gail Model and It’s Predictors in Saudi Women. Asian Pac J Cancer Prev. 2017 Nov 26; 18(11): 2971-2975. | Search |
| 1. Banegas 2012 | Banegas MP, Gail MH, LaCroix A, Thompson B, Martinez ME, Wactawski-Wende J, John EM, Hubbell FA, Yasmeen S, Katki HA. Evaluating breast cancer risk projections for Hispanic women. Breast Cancer Res Treat. 2012 Feb; 132(1): 347-53. | Search |
| 1. Bener 2017 | Bener A, Çatan F, El Ayoubi HR, Acar A, Ibrahim WH. Assessing Breast Cancer Risk Estimates Based on the Gail Model and Its Predictors in Qatari Women. J Prim Care Community Health. 2017 Jul; 8(3): 180-187. | Search |
| 1. Boggs 2013 | Boggs DA, Rosenberg L, Pencina MJ, Adams-Campbell LL, Palmer JR. Validation of a breast cancer risk prediction model developed for Black women. J Natl Cancer Inst. 2013 Mar 6; 105(5): 361-7. | Search |
| 1. Brentnall 2015 | Brentnall AR, Harkness EF, Astley SM, Donnelly LS, Stavrinos P, Sampson S, Fox L, Sergeant JC, Harvie MN, Wilson M, Beetles U, Gadde S, Lim Y, Jain A, Bundred S, Barr N, Reece V, Howell A, Cuzick J, Evans DG. Mammographic density adds accuracy to both the Tyrer-Cuzick and Gail breast cancer risk models in a prospective UK screening cohort. Breast Cancer Res. 2015 Dec 1; 17(1): 147. | Search |
| 1. Buron 2013 | Buron A, Vernet M, Roman M, Checa MA, Pérez JM, Sala M, Comas M, Murta-Nascimiento C, Castells X, Macià F. Can the Gail model increase the predictive value of a positive mammogram in a European population screening setting? Results from a Spanish cohort. Breast. 2013 Feb; 22(1): 83-8. | Search |
| 1. Chay 2012 | Chay WY, Ong WS, Tan PH, Jie Leo NQ, Ho GH, Wong CS, Chia KS, Chow KY, Tan M Sr, Ang P Sr. Validation of the Gail model for predicting individual breast cancer risk in a prospective nationwide study of 28,104 Singapore women. Breast Cancer Res. 2012 Jan 30; 14(1): R19. | Search |
| 1. Dartois 2015 | Dartois L, Gauthier É, Heitzmann J, Baglietto L, Michiels S, Mesrine S, Boutron-Ruault MC, Delaloge S, Ragusa S, Clavel-Chapelon F, Fagherazzi G. A comparison between different prediction models for invasive breast cancer occurrence in the French E3N cohort. Breast Cancer Res Treat. 2015 Apr; 150(2): 415-26. | Search |
| 1. Erbil 2015 | Erbil N, Dundar N, Inan C, Bolukbas N. Breast cancer risk assessment using the Gail model: a Turkish study. Asian Pac J Cancer Prev. 2015; 16(1): 303-6. | Search |
| 1. Ewaid 2017 | Ewaida SH, Al-Azzawib LHA. Breast cancer risk assessment by Gail Model in women of Baghdad. Alexandria Journal of Medicine. 53, 2, 2017, 183-186 | Search |
| 1. Gail 2015b | Gail MH. Twenty-five years of breast cancer risk models and their applications. J Natl Cancer Inst. 2015 Feb 26; 107(5). pii: djv042. | Search |
| 1. Gao 2012 | Gao F, Machin D, Chow KY, Sim YF, Duffy SW, Matchar DB, Goh CH, Chia KS. Assessing risk of breast cancer in an ethnically South-East Asia population (results of a multiple ethnic groups study). BMC Cancer. 2012 Nov 19; 12: 529. | Search |
| 1. Ghoncheh 2017 | Ghoncheh M, Ziaee F, Karami M, Poorolajal J. Validating the IBIS and BOADICEA Models for Predicting Breast Cancer Risk in the Iranian Population. Clin Breast Cancer. 2017 Jun; 17(3): e113-e118. | Search |
| 1. Khazaee-Pool 2016 | Khazaee-Pool M, Majlessi F, Nedjat S, Montazeri A, Janani L, Pashaei T. Assessing Breast Cancer Risk among Iranian Women Using the Gail Model. Asian Pac J Cancer Prev. 2016; 17(8): 3759-62. | Search |
| 1. MacInnis 2013 | MacInnis RJ, Bickerstaffe A, Apicella C, Dite GS, Dowty JG, Aujard K, Phillips KA, Weideman P, Lee A, Terry MB, Giles GG, Southey MC, Antoniou AC, Hopper JL. Prospective validation of the breast cancer risk prediction model BOADICEA and a batch-mode version BOADICEA Centre. Br J Cancer. 2013 Sep 3; 109(5): 1296-301. | Search |
| 1. Mazzola 2014 | Mazzola E, Chipman J, Cheng SC, Parmigiani G. Recent BRCAPRO upgrades significantly improve calibration. Cancer Epidemiol Biomarkers Prev. 2014 Aug; 23(8): 1689-95. | Search |
| 1. Mirghafourvand 2016 | Mirghafourvand M, Mohammad-Alizadeh-Charandabi S, Ahmadpour P, Rahi P. Breast Cancer Risk Based on the Gail Model and its Predictors in Iranian Women. Asian Pac J Cancer Prev. 2016; 17(8): 3741-5. | Search |
| 1. Park 2013 | Park B, Ma SH, Shin A, Chang MC, Choi JY, Kim S, Han W, Noh DY, Ahn SH, Kang D, Yoo KY, Park SK. Korean risk assessment model for breast cancer risk prediction. PLoS One. 2013 Oct 25; 8(10): e76736. | Search |
| 1. Pastor-Barriuso 2013 | Pastor-Barriuso R, Ascunce N, Ederra M, Erdozáin N, Murillo A, Alés-Martínez JE, Pollán M. Recalibration of the Gail model for predicting invasive breast cancer risk in Spanish women: a population-based cohort study. Breast Cancer Res Treat. 2013 Feb; 138(1): 249-59. | Search |
| 1. Petracci 2011 | Petracci E, Decarli A, Schairer C, Pfeiffer RM, Pee D, Masala G, Palli D, Gail MH. Risk factor modification and projections of absolute breast cancer risk. J Natl Cancer Inst. 2011 Jul 6; 103(13): 1037-48. | Search |
| 1. Seyednoori 2012 | Seyednoori T, Pakseresht S, Roushan Z. Risk of developing breast cancer by utilizing Gail model. Women Health. 2012; 52(4): 391-402. | Search |
| 1. Stahlbom 2012 | Ståhlbom AK, Johansson H, Liljegren A, von Wachenfeldt A, Arver B. Evaluation of the BOADICEA risk assessment model in women with a family history of breast cancer. Fam Cancer. 2012 Mar; 11(1): 33-40. | Search |
| 1. Ulusoy 2010 | Ulusoy C, Kepenekci I, Kose K, Aydintug S, Cam R. Applicability of the Gail model for breast cancer risk assessment in Turkish female population and evaluation of breastfeeding as a risk factor. Breast Cancer Res Treat. 2010 Apr; 120(2): 419-24. | Search |
| **Secondary publications (did not report the original risk prediction model), N= 18** | | |
| 1. Amir 2010 | Amir E, Freedman OC, Seruga B, Evans DG. Assessing women at high risk of breast cancer: a review of risk assessment models. J Natl Cancer Inst 2010 May 19; 102(10): 680-91. | Search |
| 1. Arrospide 2013 | Arrospide A, Forne C, Rue M, Tora N, Mar J, Bare M. An assessment of existing models for individualized breast cancer risk estimation in a screening program in Spain. BMC Cancer 2013 Dec 10; 13: 587 | Search |
| 1. Barke 2017 | Barke LD, Freivogel ME. Breast Cancer Risk Assessment Models and High-Risk Screening. Radiol Clin North Am 2017 May; 55(3): 457-74. | Search |
| 1. Biswas 2013 | Biswas S, Atienza P, Chipman J, Hughes K, Barrera AM, Amos CI, et al. Simplifying clinical use of the genetic risk prediction model BRCAPRO. Breast Cancer Res Treat 2013 Jun; 139(2): 571-9. | Search |
| 1. Collins 2016 | Collins IM, Bickerstaffe A, Ranaweera T, Maddumarachchi S, Keogh L, Emery J, et al. iPrevent(R): a tailored, web-based, decision support tool for breast cancer risk assessment and management. Breast Cancer Res Treat 2016 Feb; 156(1): 171-82. | Search |
| 1. Dent 2012 | Dent TH, Wright CF, Stephan BC, Brayne C, Janssens AC. Risk prediction models: a framework for assessment. Public Health Genomics. 2012; 15(2): 98-105. | Search |
| 1. Engel 2015 | Engel C, Fischer C. Breast cancer risks and risk prediction models. Breast Care (Basel) 2015 Feb; 10(1): 7-12. | Search |
| 1. Gail 2015a | Gail MH, Pfeiffer RM. Is the Benign Breast Disease Breast Cancer Model Well Calibrated? J Clin Oncol. 2015 Sep 1; 33(25): 2829-30. | Search |
| 1. Gong 2014 | Gong G, Quante AS, Terry MB, Whittemore AS. Assessing the goodness of fit of personal risk models. Stat Med 2014 Aug 15; 33(18): 3179-90. | Search |
| 1. Green 2013 | Green VL. Breast cancer risk assessment, prevention, and the future. Obstet Gynecol Clin North Am 2013 Sep; 40(3): 525-49. | Search |
| 1. Lee AJ 2014 | Lee AJ, Cunningham AP, Kuchenbaecker KB, Mavaddat N, Easton DF, Antoniou AC; Consortium of Investigators of Modifiers of BRCA1/2; Breast Cancer Association Consortium. BOADICEA breast cancer risk prediction model: updates to cancer incidences, tumour pathology and web interface. Br J Cancer. 2014 Jan 21; 110(2): 535-45. | Search |
| 1. Lee JY 2014 | Lee JY, Klimberg S, Bondurant KL, Phillips MM, Kadlubar SA. Cross-sectional study to assess the association of population density with predicted breast cancer risk. Breast J. 2014 Nov-Dec; 20(6): 615-21. | Search |
| 1. Park 2015; | Park KY, Li G, Platt MO. Monocyte-derived macrophage assisted breast cancer cell invasion as a personalized, predictive metric to score metastatic risk. Sci Rep 2015 Sep 9; 5: 13855. | Search |
| 1. Prado 2010; | Prado A, Andrades P, Parada F. Recent developments in the ability to predict and modify breast cancer risk. J Plast Reconstr Aesthet Surg. 2010 Oct; 63(10): 1581-7. | Search |
| 1. Schonfeld 2010; | Schonfeld SJ, Bhatti P, Brown EE, Linet MS, Simon SL, Weinstock RM, et al. Polymorphisms in oxidative stress and inflammation pathway genes, low-dose ionizing radiation, and the risk of breast cancer among US radiologic technologists. Cancer Causes Control 2010 Nov; 21(11): 1857-66. | Search |
| 1. Stegeman 2012 | Stegeman I, Bossuyt PM. Cancer risk models and preselection for screening. Cancer Epidemiol. 2012 Oct; 36(5): 461-9. | Search |
| 1. Varesco 2013 | Varesco L, Viassolo V, Viel A, Gismondi V, Radice P, Montagna M, Alducci E, Della Puppa L, Oliani C, Tommasi S, Caligo MA, Vivanet C, Zuradelli M, Mandich P, Tibiletti MG, Cavalli P, Lucci Cordisco E, Turchetti D, Boggiani D, Bracci R, Bruzzi P, Bonelli L. Performance of BOADICEA and BRCAPRO genetic models and of empirical criteria based on cancer family history for predicting BRCA mutation carrier probabilities: a retrospective study in a sample of Italian cancer genetics clinics. Breast. 2013 Dec; 22(6): 1130-5. | Search |
| 1. Wang 2014 | Wang P, Wang Y, Yan H, Xie Q, Zhao L, Xu S, et al. Genetic variation in the major mitotic checkpoint genes and risk of breast cancer: a multigenic study on cancer susceptibility. Tumour Biol 2014 Jul; 35(7): 6701-5. | Search |
| **Wrong setting, population (did not assess average risk women), N= 8** | | |
| 1. Boughey 2010 | Boughey JC, Hartmann LC, Anderson SS, Degnim AC, Vierkant RA, Reynolds CA, Frost MH, Pankratz VS. Evaluation of the Tyrer-Cuzick (International Breast Cancer Intervention Study) model for breast cancer risk prediction in women with atypical hyperplasia. J Clin Oncol 2010 Aug 1; 28(22): 3591-6. | Search |
| 1. Eoh 2017 | Eoh KJ, Park JS, Park HS, Lee ST, Han J, Lee JY, et al. BRCA1 and BRCA2 mutation predictions using the BRCAPRO and Myriad models in Korean ovarian cancer patients. Gynecol Oncol 2017 Apr; 145(1): 137-41. | Search |
| 1. Hiatt 2014 | Hiatt RA, Porco TC, Liu F, Balke K, Balmain A, Barlow J, et al. A multilevel model of postmenopausal breast cancer incidence. Cancer Epidemiol Biomarkers Prev 2014 Oct; 23(10): 2078-92. | Search |
| 1. Locke 2016 | Locke PA, Weil MM. Personalized cancer risk assessments for space radiation exposures. Front Oncol. 2016 Feb 22; 6: 38. | Search |
| 1. Pankratz 2015 | Pankratz VS, Degnim AC, Frank RD, Frost MH, Visscher DW, Vierkant RA, et al. Model for individualized prediction of breast cancer risk after a benign breast biopsy. J Clin Oncol 2015 Mar 10; 33(8): 923-9. | Search |
| 1. Schonberg 2016 | Schonberg MA, Li VW, Eliassen AH, Davis RB, LaCroix AZ, McCarthy EP, et al. Performance of the Breast Cancer Risk Assessment Tool Among Women Age 75 Years and Older. J Natl Cancer Inst 2016 Mar; 108(3). | Search |
| 1. Tamimi 2010 | Tamimi RM, Rosner B, Colditz GA. Evaluation of a breast cancer risk prediction model expanded to include category of prior benign breast disease lesion. Cancer. 2010 Nov 1; 116(21): 4944-53. | Search, + Syst. Review [Anothaisintawee2012] |
| 1. Tice 2005 | Tice JA, Miike R, Adduci K, Petrakis NL, King E, Wrensch MR. Nipple aspirate fluid cytology and the Gail model for breast cancer risk assessment in a screening population. Cancer Epidemiol Biomarkers Prev. 2005 Feb; 14(2): 324-8. | Search, + Syst. Review [Anothaisintawee2012] |
| **Not risk prediction model (did not report absolute risk, only relative risks or risk scores), N= 6** | | |
| 1. Anothaisintawee 2014 | Anothaisintawee T, Teerawattananon Y, Wiratkapun C, Srinakarin J, Woodtichartpreecha P, Hirunpat S, Wongwaisayawan S, Lertsithichai P, Kasamesup V, Thakkinstian A. Development and validation of a breast cancer risk prediction model for Thai women: a cross-sectional study. Asian Pac J Cancer Prev. 2014; 15(16): 6811-7. | Search |
| 1. Hsieh 2017 | Hsieh YC, Tu SH, Su CT, Cho EC, Wu CH, Hsieh MC, et al. A polygenic risk score for breast cancer risk in a Taiwanese population. Breast Cancer Res Treat 2017 May; 163(1): 131-8. | Search |
| 1. Lee CP 2014 | Lee CP, Irwanto A, Salim A, Yuan JM, Liu J, Koh WP, Hartman M.  Breast cancer risk assessment using genetic variants and risk factors in a Singapore Chinese population. Breast Cancer Res. 2014 Jun 18; 16(3): R64. | Search |
| 1. Lophatananon 2017 | Lophatananon A, Usher-Smith J, Campbell J, Warcaba J, Silarova B, Waters EA, et al. Development of a Cancer Risk Prediction Tool for Use in the UK Primary Care and Community Settings. Cancer Prev Res (Phila) 2017 Jul; 10(7): 421-30. | Search |
| 1. Tesic 2013; | Tesic V, Kolaric B, Znaor A, Kuna SK, Brkljacic B. Mammographic density and estimation of breast cancer risk in intermediate risk population. Breast J. 2013 Jan-Feb;19(1):71-8. | Search |
| 1. Zheng 2012 | Zheng B, Sumkin JH, Zuley ML, Wang X, Klym AH, Gur D. Bilateral mammographic density asymmetry and breast cancer risk: a preliminary assessment. Eur J Radiol 2012 Nov; 81(11): 3222-8. | Search |
| **Wrong study outcomes (did not assess longitudinal risk, only transversal/observed risk), N= 1** | | |
| 1. Lee 2004 | Lee EO, Ahn SH, You C, Lee DS, Han W, Choe KJ, Noh DY. Determining the main risk factors and high-risk groups of breast cancer using a predictive model for breast cancer risk assessment in South Korea. Cancer Nurs. 2004 Sep-Oct; 27(5): 400-6. | Search, + Syst. Review [Anothaisintawee2012] |
| **Wrong study outcomes (assessed risk of breast cancer relapse), N= 1** | | |
| 1. Lee JM 2015 | Lee JM, Buist DS, Houssami N, Dowling EC, Halpern EF, Gazelle GS, Lehman CD, Henderson LM, Hubbard RA. Five-year risk of interval-invasive second breast cancer. J Natl Cancer Inst. 2015 Apr 22; 107(7). | Search |

**Supplementary table 3. Extended characteristics of the included studies**

| **Study ID** | **Journal** | **Country** | **Target population** | **Risk model** | | | | | | | |
| --- | --- | --- | --- | --- | --- | --- | --- | --- | --- | --- | --- |
|  |  |  |  | **Model ID** | **Design used for collecting the inputs** | **Method** | **Sample size** | **Breast cancer type** | **Risk factors (categories)** | **Discriminatory accuracy^a^** | **Calibration^a^** |
| **Breast Cancer Risk Assessment Tool ‘BCRAT’ Model** | | | | | | | | | | | |
| 1. Gail 1989 | J Natl Cancer Inst | USA | Caucasian women aged 20 to 79 years | Original | Nested case-control | Logistic regression | 2,852 cases and 3,146 controls | Invasive and in situ carcinomas | Age (2), Menarche (3), Previous biopsies (3), Age at first birth (4), First degree breast cancer family history (3) | None | None |
| 1. Banegas 2017 | J Natl Cancer Inst | USA | Hispanic women aged 25 to 79 years | Gail 1989 | Case-control | Logistic regression | 1,086 cases and 1,411 controls | Invasive carcinomas | Age (2), Menarche (3), Previous biopsies (2), Age at first birth (3), First degree breast cancer family history (2) | AUROC: 0.56 and 0.62 for US-born and foreign-born, respectively | E/O^b^: 0.93 and 1.52 for US-born and foreign-born, respectively |
| 1. Boyle 2004 | Eur J Cancer Prev | Italy | Caucasian women aged 20 to 74 years | Gail 1989 | Case-control | Logistic regression | 2,569 cases and 2,588 controls | Invasive carcinomas | Age (n), Menarche (3), Age at first birth (3), First degree breast cancer family history (2), BMI (3), Alcohol (3), Physical activity (3), HRT (2), Diet beta-carotene/vitE (5), Diet fruits/vegetables (5) | AUROC: 0.60 | E/O^b^: 1.03 |
| 1. Chen 2006 | J Natl Cancer Inst | USA | Caucasian women aged 35 to 74 years | Gail 1989 | Nested case-control | Logistic regression | 1,280 cases and 4,035 controls | Invasive and in situ carcinomas | Age (2), Weight (6), Breast density (5), Menarche (3), Previous biopsies (3), Age at first birth (4), First degree breast cancer family history (3), Atypical hyperplasia (2) | None | None |
| 1. Decarli 2006 | J Natl Cancer Inst | Italy | Caucasian women aged 20 to 74 years | Gail 1989 | Case-control | Logistic regression | 2,569 cases and 2,588 controls | Invasive carcinomas | Age (2), Menarche (3), Previous biopsies (3), Age at first birth (4), First degree breast cancer family history (3) | AUROC: 0.59 | E/O: 0.96 |
| 1. Gail 2007 | J Natl Cancer Inst | USA | African-american women aged 35 to 64 years | Gail 1989 | Case-control | Logistic regression | 1,607 cases and 1,647 controls | Invasive carcinomas | Age (2), Menarche (3), Previous biopsies (3), Age at first birth (4), First degree breast cancer family history (3) | AUROC: 0.56 | E/O^b^: 0.93 |
| 1. Matsuno 2011 | J Natl Cancer Inst | USA | Asian women aged 20 to 55 years | Gail 1989 | Case-control | Logistic regression | 589 cases and 952 controls | Invasive carcinomas | Age (2), Menarche (3), Previous biopsies (3), Age at first birth (4), First degree breast cancer family history (n), Ethnicity (6) | AUROC: 0.61 | E/O^b^: 0.85 |
| 1. Novotny 2006 | Breast Cancer Res Treat | Czech Republic | Multiple ethnic women aged 23 to 84 years | Gail 1989 | Case-control | Logistic regression | 2,299 cases and 2,299 controls | Invasive carcinomas | Age (2), Menarche (3), Previous biopsies (3), Age at first birth (4), First degree breast cancer family history (3), First degree family history of cancer (5), Parity (n), Breast inflammatory disease (2) | None | None |
| 1. Tice 2005 | Breast Cancer Res Treat | USA | Multiple ethnic women older than 35 years | Gail 1989 | Cohort | Cox proportional-hazards model | 955 cases, 81,777 women | Invasive and in situ carcinomas | Age (2), Menarche (3), Previous biopsies (3), Age at first birth (4), First degree breast cancer family history (3), Breast density (4) | AUROC: 0.68 | None |
| 1. Zhang 2018 | Plos Medicine | USA | Caucasian women aged 30 to 64 years | BCRAT and Rosner and Colditz model | Nested case-control | Logistic regression | 4,006 cases, 7,874 controls | Invasive Carcinoma | Age (2), Menarche (3), Previous biopsies (3), Age at first birth (4), Breast Cancer family history (2), Polygenic Risk Score(n), Mammographic density (n), Estrone Sulfate (n), Testosterone (n), Prolactin (n) | AUROC: 0.65 | None |
| **Breast Cancer Surveillance Consortium ‘BCSC’ model** | | | | | | | | | | | |
| 1. Tice 2008 | Ann Intern Med | USA | Multiple ethnic women aged 35 to 84 years | Gail 1989/BCS model | Cohort | Cox proportional-hazards model | 14,776 cases, 1,095,484 women | Invasive carcinomas | Age (n), Ethnicity (4), First degree breast cancer family history (2), Previous biopsies (2), Breast density (BIRADS) | AUROC: 0.66 | E/O: 1.03 |
| 1. Kerlikowske 2015 | Cancer Epidemiol Biomarkers Prev | USA | Multiple ethnic women aged 35 to 74 years | BCSC model | Cohort | Cox proportional-hazards model | 13,715 cases, 722,654 women | Invasive carcinomas | Age (n), Ethnicity (6), First degree breast cancer family history (2), Previous biopsies (2), Changes in breast density (16) | AUROC: 0.64 | 5-years E/O: 0.98, 10-years E/O: 0.95 |
| 1. Tice 2015 | J Clin Oncol | USA | Multiple ethnic women aged 35 to 74 years | BCSC model | Cohort | Cox proportional-hazards model | 17,908 cases, 1,135,977 women | Invasive carcinomas | Age (n), Ethnicity (4), First degree breast cancer family history (2), Breast density (4), Benign breast disease (6) | AUROC: 0.67 | 5-years E/O: 1.04, 10-years E/O: 1.05 |
| 1. Vachon 2015 | J Natl Cancer Inst | USA | Multiple ethnic women. Age was not specified | BCSC model | Nested case-control | Logistic regression | 1643 cases and 2397 controls | Invasive carcinomas | Age (n), Ethnicity (6), First degree breast cancer family history (2), Previous biopsies (2), Breast density (4), Polygenetic risk score (n) | AUROC: 0.69 | None |
| 1. Shieh 2016 | Breast Cancer Res Treat | USA | Multiple ethnic women. Age was not specified | BCSC model | Nested case-control | Logistic regression | 495 cases and 486 controls | Invasive carcinomas | Age (n), Ethnicity (6), First degree breast cancer family history (2), Previous biopsies (2), Breast density (4), Polygenetic risk score (n), BMI (n) | AUROC: 0.65 | None |
| **Rosner and Colditz model based on the ‘Nurse Health’s Study’** | | | | | | | | | | | |
| 1. Rosner 1996 | J Natl Cancer Inst | USA | Caucasian women aged 30 to 64 yers | Original | Prospective cohort | Poisson regression model | 2,249 cases, 89,132 women | Invasive carcinomas | Age (n), Menarche (n), Age at first birth (n), Menopause (n), Age at subsequent births (n) | None | None |
| 1. Colditz 2000 | Am J Epidemiol | USA | Caucasian women aged 30 to 64 yers | Rosner and Colditz model | Prospective cohort | Poisson regression model | 1,761 cases, 58,520 women | Invasive carcinomas | Age (n), Menarche (n), Age at first birth (n), Menopause (n), Age at subsequent births (n), Benign breast disease (2), HRT (n), First degree breast cancer family history (2), Weight (n), BMI (n), Alcohol (n) | None | None |
| 1. Colditz 2004 | J Natl Cancer Inst | USA | Caucasian women aged 30 to 64 yers | Rosner and Colditz model | Prospective cohort | Poisson regression model | 2,096 cases, 66,145 women | Invasive carcinomas | Age (n), Menarche (n), Age at first birth (n), Menopause (n), Age at subsequent births (n), Benign breast disease (2), HRT (n), First degree breast cancer family history (2), Weight (n), BMI (n), Alcohol (n) | AUROC: 0.64 and 0.61 for ER+/PR+ and ER-/PR- tumours, respectively | None |
| 1. Rosner 2008 | Breast Cancer Res | USA | Caucasian women aged 30 to 64 yers | Rosner and Colditz model | Prospective cohort | Poisson regression model | 1,559 cases, 59,812 women | Invasive carcinomas | Age (n), Menarche (n), Age at first birth (n), Menopause (n), Age at subsequent births (n), Benign breast disease (2), HRT (n), First degree breast cancer family history (2), Weight (n), BMI (n), Alcohol (n), Estradiol levels (n) | AUROC: 0.65 | None |
| 1. Zhang 2018 | Plos Medicine | USA | Caucasian women aged 30 to 64 yers | BCRAT and Rosner and Colditz model | Nested case-control | Logistic regression | 4,006 cases, 7,874 controls | Invasive Carcinoma | ge (n), Menarche (n), Age at first birth (n), Menopause (n), Age at subsequent births (n), Benign breast disease (2), HRT (n), First degree breast cancer family history (2), Weight (n), BMI (n), Alcohol (n), Early life somatotype, Polygenic Risk Score(n), Mammographic density (n), Estrone Sulfate (n), Testosterone (n), Prolactin (n) | AUROC: 0.68 | None |
| **International Breast Cancer Intervention Study ‘IBIS’ model** | | | | | | | | | | | |
| 1. Tyrer 2004 | Stats Med | UK | Multiple ethnic women. Not specified age | Original | Systematic review | Relative risk values are collected from studies included in the systematic review | NA | Invasive carcinomas | Age (n), Gen phenotype (6), Family history (n, relationship, age), Menarche (n), Age at first birth (5), Menopause (n), Atypical Hyperplasia (2), Lobular carcinoma in situ (2), Height (3), BMI (5) | None | None |
| **Other** | | | | | | | | | | | |
| 1. Barlow 2006 | J Natl Cancer Inst | USA | Multiple ethnic women aged 35 to 84 years | Original | Prospective cohort | Logistic regression | 11,638 cases, 2,392,988 mammograms | Invasive and in situ carcinomas | Age (9), Age at first birth (4), Ethnicity (6), Menopause (2), First degree breast cancer family history (5), Previous biopsies (4), Breast density (5), HRT (3), BMI (5), Previous false positive or true negative screen result (2), Menopausal status (3) | AUROC: 0.63 and 0.62 for pre and post-menopausal status | E/O: 1.00 and 1.01 for pre and post-menopausal status |
| 1. Eriksson 2017 | Breast Cancer Res | Sweden | Caucasian women aged 40 to 74 years | Original | Nested case-control | Cox proportional-hazards model by Individualized  Coherent Absolute Risk Estimator (iCARE). | 433 cases and 1,732 controls | Invasive and in situ carcinomas | Age (7), BMI (n), HRT (2), Breast cancer family history (2), Menopause (2), Breast density (4), Microcalcifications (5), Mases (n) | AUROC: 0.71 | None |
| 1. Ueda 2003 | Breast Cancer | Japan | Asian women. Age was not specified. | Original | Case-control | Logistic regression | 376 cases and 430 controls | Invasive carcinomas | Age (n), Menarche (3), Age at first birth (5), BMI (2), Breast cancer family history (2) | None | None |
| 1. Wang 2014 | Tumour Biol | China | Asian women aged 35 to 70 years | Original | Systematic review | Relative risk values are collected from studies included in the systematic review | NA | Invasive carcinomas | Age (7), Menarche (2), Previous biopsies (2), Age at first birth (2), First degree breast cancer family history (2), Breastfeeding (2), Abortion (2) | AUROC: 0.64 | None |

^a^ Discriminatory accuracy and prediction accuracy values represents the statistics published in the original articles for the general population. Subgroup values are not reported here. ^b^ The original publication reported the Observed/Expected ratio. E/O ratios were calculated based on the original information. AUROC: Area under the receiver operating characteristic curve. NA: Not applicable. E/O: Expected/observed ratio. BMI: Body mass index. HRT: Hormone replacement therapy. BIRADS: Breast Imaging Reporting and Data System. ER+: Oestrogen receptor positive. PR+: Progesterone receptor positive.

**Supplementary table 4. Detailed appraisal and judgements for the risk of bias assessment**

| **Study ID** | **Validation** | **Design** | **Data** | **Analysis** | **Reporting** | **Interpretation** | **Conflict** |
| --- | --- | --- | --- | --- | --- | --- | --- |
| **Breast Cancer Risk Assessment Tool ‘BCRAT’** | | | | | | | |
| Banegas 2017 | The AUC value and the E/O ratio were estimated and reported in the study. Externally validated. | A case control design was used to obtain the risk estimations instead of a prospective/retrospective study. A case-control study does not take into account the time into the analysis and therefore this is a relevant limitation for the estimation of individualized future risk. | More than 50% of the risk factors information included in the model was self-reported. | Logistic regression model that did not take into account the time seems not as adequate as a Cos or a Poisson model. | The model is adequately documented and reported, including details of model development, data sources, and funding sources. | The interpretation of the results of the model seems fair and balanced | The authors declare no conflict of interests |
| Boyle 2004 | The AUC value and the E/O ratio were estimated and reported in the study. Also the results were compared with other model (Gail 1989). No external validation. | A case control design was used to obtain the risk estimations instead of a prospective/retrospective study. A case-control study does not take into account the time into the analysis and therefore this is a relevant limitation for the estimation of individualized future risk. | More than 50% of the risk factors information included in the model was self-reported. | Logistic regression model that did not take into account the time seems not as adequate as a Cos or a Poisson model. | The model is adequately documented and reported, including details of model development, data sources, and funding sources. | The interpretation of the results of the model seems fair and balanced | Not reported |
| Chen 2006 | The results were compared with other model (Gail 1989) but this study did not report validation measures such as E/O ratio, AUC, or internal and external validation process. | A case control design was used to obtain the risk estimations instead of a prospective/retrospective study. A case-control study does not take into account the time into the analysis and therefore this is a relevant limitation for the estimation of individualized future risk. | More than 50% of the risk factors information included in the model was self-reported. | Logistic regression model that did not take into account the time seems not as adequate as a Cos or a Poisson model. | The model is adequately documented and reported, including details of model development, data sources, and funding sources. | The interpretation of the results of the model seems fair and balanced | Not reported |
| Decarli 2006 | The AUC value and the E/O ratio were estimated and reported in the study. Externally validated. | A case control design was used to obtain the risk estimations instead of a prospective/retrospective study. A case-control study does not take into account the time into the analysis and therefore this is a relevant limitation for the estimation of individualized future risk. | More than 50% of the risk factors information included in the model was self-reported. | Logistic regression model that did not take into account the time seems not as adequate as a Cos or a Poisson model. | The model is adequately documented and reported, including details of model development, data sources, and funding sources. | The interpretation of the results of the model seems fair and balanced | Not reported |
| Gail 1989 | This study did not report validation measures such as E/O ratio, AUC, or internal and external validation process. | A case control design was used to obtain the risk estimations instead of a prospective/retrospective study. A case-control study does not take into account the time into the analysis and therefore this is a relevant limitation for the estimation of individualized future risk. | More than 50% of the risk factors information included in the model was self-reported. | Logistic regression model that did not take into account the time seems not as adequate as a Cos or a Poisson model. | The model is adequately documented and reported, including details of model development, data sources, and funding sources. | The article is not objective with its limitations and gives little information about them. | Not reported |
| Gail 2007 | The AUC value and the E/O ratio were estimated and reported in the study. Externally validated. | A case control design was used to obtain the risk estimations instead of a prospective/retrospective study. A case-control study does not take into account the time into the analysis and therefore this is a relevant limitation for the estimation of individualized future risk. | More than 50% of the risk factors information included in the model was self-reported. | Logistic regression model that did not take into account the time seems not as adequate as a Cos or a Poisson model. | The model is adequately documented and reported, including details of model development, data sources, and funding sources. | The interpretation of the results of the model seems fair and balanced | Not reported; funded by National Institute of Child Health and Human Development and National Cancer Institute (USA) |
| Matsuno 2011 | The AUC value and the E/O ratio were estimated and reported in the study. External validation was conducted using WHI data. | A case control design was used to obtain the risk estimations instead of a prospective/retrospective study. A case-control study does not take into account the time into the analysis and therefore this is a relevant limitation for the estimation of individualized future risk. | More than 50% of the risk factors information included in the model was self-reported. Missing-data imputation method was used for race when unknown. | Logistic regression model that did not take into account the time seems not as adequate as a Cos or a Poisson model. | The model is adequately documented and reported, including details of model development, data sources, and funding sources. | Balanced interpretation with extensive discussion of limitations | Not reported; funded by National Cancer Institute (USA) |
| Novotny 2006 | This study reported a prospective validation of the Gail model and the development of a new Czech model for which did not report validation measures. | A case control design was used to obtain the risk estimations instead of a prospective/retrospective study. A case-control study does not take into account the time into the analysis and therefore this is a relevant limitation for the estimation of individualized future risk. | More than 50% of the risk factors information included in the model was self-reported. Data was taken from a national cancer registry and survey of 15,694 women (14, 566 responses) who underwent screening mammography. | Logistic regression model that did not take into account the time seems not as adequate as a Cos or a Poisson model. | The model is adequately documented and reported, including details of model development, data sources, and funding sources. | The interpretation of the results of the model seems fair and balanced | Not reported |
| Tice 2005 | The AUC value was estimated and reported in the study. No external validation. | Retrospective cohort design is appropriate for purpose of estimating future risk. | More than 50% of the risk factors information included in the model was self-reported. In addition missing data in the variable race were inputted. | Cox proportional hazard risk model seems adequate | The proportion of the population that was used for the development of the model and for its validation is not clearly reported | The interpretation of the results of the model seems fair and balanced | Not reported; funded by National Cancer Institute (USA) and the Building Interdisciplinary Research Careers in Women's Health. |
| Zhang 2018 | The AUC value were estimated and reported in the study and compared with the Gail 1989 and Rosner-Colditz model. No external validation. | Retrospective cohort design is appropriate for purpose of estimating future risk. Case-control design was used for the SNPs and MD information which might be appropriate since this information may not dramatically depend on exposure time. | Although Nurses' Health Study cohort seems reasonable data source with suitable exclusions made for data quality, more than 50% of the risk factors was self-reported. | Logistic regression model that did not take into account the time seems not as adequate as a Cos or a Poisson model. | Technical and nontechnical documentation appear adequate | Limitations and implications of model seem fairly discussed | The authors declare no conflict of interests |
| **Breast Cancer Surveillance Consortium ‘BCSC’ model** | | | | | | | |
| Kerlikowsky 2015 | The AUC value and the E/O ratio were estimated and reported in the study. No external validation. | Retrospective cohort design is appropriate for purpose of estimating future risk. | Less than 50% of the risk factors information included in the model was self-reported | Cox proportional hazard risk model seems adequate | The proportion of the population that was used for the development of the model and for its validation is not clearly reported | The interpretation of the model is appropriately modest. | The authors declare no conflict of interests |
| Shieh 2016 | The AUC was estimated and reported in the study. Also the results were compared with other study assessing the same model (Tice 2008). No external validation. | Retrospective cohort design is appropriate for purpose of estimating future risk. Case-control design was used for the SNPs information which might be appropriate since this information may not dramatically depended on exposure time. | Less than 50% of the risk factors information included in the model was self-reported | Logistic regression model that did not take into account the time seems not as adequate as a Cos or a Poisson model. | The reporting of model development lacks detail but is sufficient to understand the approach. | The interpretation seems somewhat unbalanced as it does not address the fact that the AUCs of all three risk models tested have overlapping Cis, nor does it consider cost or availability of the PRS | COI declared; one author has a minor competing interest (consultancy fees relating to contrast-enhanced mammography) |
| Tice 2008 | The E/O ratio was estimated and reported in the study. No external validation. | Retrospective cohort design is appropriate for purpose of estimating future risk. | Less than 50% of the risk factors information included in the model was self-reported | Cox proportional hazard risk model seems adequate | The reporting of model development lacks detail but is sufficient to understand the approach. | The interpretation is balanced, recognising modest discriminatory accuracy and modest improvement compared to the Gail model. The modest reproducibility of BIRADS measurement is noted. | Role of funding source and potential COI disclosed |
| Tice 2015 | The AUC value and the E/O ratio were estimated and reported in the study. Also the results were compared with other study assessing the same model (Tice 2008)). No external validation. | Retrospective cohort design is appropriate for purpose of estimating future risk. | Less than 50% of the risk factors information included in the model was self-reported | Cox proportional hazard risk model seems adequate | The proportion of the population that was used for the development of the model and for its validation is not clearly reported | The interpretation emphasises the superiority over other models, gives little information on limitations, and offers limited context from the empirical literature. | The authors declare no conflict of interests |
| Vachon 2015 | The AUC value was estimated and reported in the study. Also the AUC was compared with other study assessing the same model (Tice 2008). No external validation. | Retrospective cohort design is appropriate for purpose of estimating future risk. Case-control design was used for the SNPs and MD information which might be appropriate since this information may not dramatically depend on exposure time. | Less than 50% of the risk factors information included in the model was self-reported | Logistic regression model that did not take into account the time seems not as adequate as a Cos or a Poisson model. | The model is adequately documented and reported, including details of model development, data sources, and funding sources | The interpretation seems somewhat unbalanced as it does not address the fact that the AUCs on this paper and on the previous model which it compares (Tice 2008) overlap CIS so they are not significantly different and this should be mentioned. | Not reported |
| **Rosner and Colditz model based on the ‘Nurse Health’s Study’** | | | | | | | |
| Colditz 2000 | This study did not report validation measures such as E/O ratio, AUC, or internal and external validation process. It was externally validated in other paper (Rosner 2013) | Prospective cohort design is appropriate for purpose of estimating future risk. | Although Nurses' Health Study cohort seems reasonable data source with suitable exclusions made for data quality, more than 50% of the risk factors was self-reported. | Poisson regression model seems adequate | Technical and nontechnical documentation appear adequate | Emphasis on positive judgments of model accuracy; some discussion of limitations and inconsistencies | Not reported; funded by National Cancer Institute (USA) |
| Colditz 2004 | The AUC value was estimated and reported in the study. External validation is briefly mentioned (p. 227) against "an independent series of cases (diagnosed from 1994 through 1998)". Face validity seems acceptable and is increased by incorporation of time-varying exposure and interaction terms. | Prospective cohort design is appropriate for purpose of estimating future risk. | Although Nurses' Health Study cohort seems reasonable data source with suitable exclusions made for data quality, more than 50% of the risk factors was self-reported. | Poisson regression model seems adequate | Technical and nontechnical documentation appear adequate | Emphasis on positive judgments of model accuracy and advantages of their approach; no discussion of limitations or inconsistencies | Not reported |
| Rosner 1996 | This study estimated and reported the E/O ratio. It was externally validated in other paper (Rosner 2013) | Prospective cohort design is appropriate for purpose of estimating future risk. | Although Nurses' Health Study cohort seems reasonable data source with suitable exclusions made for data quality, more than 50% of the risk factors was self-reported. | Poisson regression model seems adequate | Technical and nontechnical documentation appear adequate | Limitations and implications of model seem fairly discussed | Not reported |
| Rosner 2008 | The AUC value was estimated and reported in the study. It was externally validated in other paper (Rosner 2013) | Prospective cohort design is appropriate for purpose of estimating future risk. | Although Nurses' Health Study cohort seems reasonable data source with suitable exclusions made for data quality, more than 50% of the risk factors was self-reported. | Poisson regression model seems adequate | Technical and nontechnical documentation appear adequate | Emphasis on positive judgments of model accuracy; some discussion of limitations and inconsistencies. The contribution of imputed estradiol to the model is probably overstated given the small change in C statistic, uncertainty due to imputation, and burden/cost of collecting blood samples. | The authors declare no conflict of interests |
| Zhang 2018 | The AUC value was estimated and reported in the study and compared with the Gail 1989 and Rosner-Colditz model. No external validation. | Retrospective cohort design is appropriate for purpose of estimating future risk. Case-control design was used for the SNPs and MD information which might be appropriate since this information may not dramatically depend on exposure time. | Although Nurses' Health Study cohort seems reasonable data source with suitable exclusions made for data quality, more than 50% of the risk factors was self-reported. | Logistic regression model that did not take into account the time seems not as adequate as a Cos or a Poisson model. | Technical and nontechnical documentation appear adequate | Limitations and implications of model seem fairly discussed | The authors declare no conflict of interests |
| **International Breast Cancer Intervention Study ‘IBIS’ model** | | | | | | | |
| Tyrer 2004 | This study did not report validation measures such as E/O ratio, AUC, or internal and external validation process. | A systematic review was used to obtain the risk estimations instead of a prospective/retrospective study. | Uses all data from other papers. | Cox proportional hazard risk model and Bayesian models to incorporate phenotype seems adequate | The model is adequately documented and reported, including details of model development, data sources, and funding sources. | Minimal interpretation but it is modest and recognises that risk estimates associated with BRCA status may be too high. | Not reported |
| **Other original models** | | | | | | | |
| Barlow 2006 | The AUC value and the E/O ratio were estimated and reported in the study. Also the results were compared with other model (Gail 1989). No external validation. | Retrospective cohort design is appropriate for purpose of estimating future risk. | More than 50% of the risk factors information included in the model was self-reported. | Logistic regression model that did not take into account the time seems not as adequate as a Cos or a Poisson model. | The model is adequately documented and reported, including details of model development, data sources, and funding sources. | The interpretation of the results of the model seems fair and balanced | The authors declare no conflict of interests |
| Eriksson 2017 | The AUC value was estimated and reported in the study. Also the results were compared with two other models (Gail 1989, Tyrer 2004). No external validation. | An anidated case-control design was used to estimate the risk factors hazard, which might be appropriate since this design may not take in to account the exposure time | Less than 50% of the risk factors information included in the model was self-reported | Uses an R package to do the estimates, this package uses Proportional hazards survival models so it seems adequate. | The model is adequately documented and reported, including details of model development, data sources, and funding sources. | The interpretation of the results of the model seems fair and balanced | The authors declare no conflict of interests |
| Ueda 2003 | This study did not report validation measures such as E/O ratio, AUC, or internal and external validation process. | A case control design was used to obtain the risk estimations instead of a prospective/retrospective study. A case-control study does not take into account the time into the analysis and therefore this is a relevant limitation for the estimation of individualized future risk. | More than 50% of the risk factors information included in the model was self-reported. General population estimate comes from a large population-based cancer registry, but otherwise data from the model come from a relatively small case-control study performed in one urban hospital. | Logistic regression model that did not take into account the time seems not as adequate as a Cos or a Poisson model. | The model is adequately documented and reported, including details of model development, data sources, and funding sources. | The interpretation of the model is appropriately modest. | Not reported. Government funded study. |
| Wang 2014 | The AUC value was estimated and reported for an independent cohort. No external validation. | A systematic review was used to obtain the risk estimations instead of a prospective/retrospective study. Most of the studies identified by the systematic review were not longitudinal and therefore not appropriate for a risk estimation. | More than 50% of the risk factors information included in the model was self-reported as it was informed by the individual studies identified by the systematic review. | Logistic regression model that did not take into account the time seems not as adequate as a Cos or a Poisson model. | The model is adequately documented and reported, including details of model development, data sources, and funding sources. | The interpretation of the model is appropriately modest. Key limitations are lack of external validation and lack of follow-up in the screened population. | The authors declare no conflict of interests |
